# Bio-Electrical Impedance Analysis: A Valid Assessment Tool for Diagnosis of Low Appendicular Lean Mass in Older Adults?

**DOI:** 10.3389/fnut.2022.874980

**Published:** 2022-06-02

**Authors:** Jantine van den Helder, Amely M. Verreijen, Carliene van Dronkelaar, Robert G. Memelink, Mariëlle F. Engberink, Raoul H. H. Engelbert, Peter J. M. Weijs, Michael Tieland

**Affiliations:** ^1^Faculty of Sports and Nutrition, Center of Expertise Urban Vitality, Amsterdam University of Applied Sciences, Amsterdam, Netherlands; ^2^Faculty of Health, Center of Expertise Urban Vitality, Amsterdam University of Applied Sciences, Amsterdam, Netherlands; ^3^Department of Nutrition and Dietetics, Amsterdam Movement Sciences, Amsterdam UMC, Vrije Universiteit Amsterdam, Amsterdam, Netherlands; ^4^Department of Rehabilitation, Amsterdam Movement Sciences, Amsterdam UMC, University of Amsterdam, Amsterdam, Netherlands

**Keywords:** aging, lean body mass, muscle, nutritional assessment, sarcopenia

## Abstract

**Background:**

The diagnosis of sarcopenia is essential for early treatment of sarcopenia in older adults, for which assessment of appendicular lean mass (ALM) is needed. Multi-frequency bio-electrical impedance analysis (MF-BIA) may be a valid assessment tool to assess ALM in older adults, but the evidences are limited. Therefore, we validated the BIA to diagnose low ALM in older adults.

**Methods:**

ALM was assessed by a standing-posture 8 electrode MF-BIA (Tanita MC-780) in 202 community-dwelling older adults (age ≥ 55 years), and compared with dual-energy X-ray absorptiometry (DXA) (Hologic Inc., Marlborough, MA, United States; DXA). The validity for assessing the absolute values of ALM was evaluated by: (1) bias (mean difference), (2) percentage of accurate predictions (within 5% of DXA values), (3) the mean absolute error (MAE), and (4) limits of agreement (Bland–Altman analysis). The lowest quintile of ALM by DXA was used as proxy for low ALM (< 22.8 kg for men, < 16.1 kg for women). Sensitivity and specificity of diagnosing low ALM by BIA were assessed.

**Results:**

The mean age of the subjects was 72.1 ± 6.4 years, with a BMI of 25.4 ± 3.6 kg/m^2^, and 71% were women. BIA slightly underestimated ALM compared to DXA with a mean bias of −0.6 ± 1.2 kg. The percentage of accurate predictions was 54% with a MAE of 1.1 kg, and limits of agreement were −3.0 to + 1.8 kg. The sensitivity for ALM was 80%, indicating that 80% of subjects who were diagnosed as low ALM according to DXA were also diagnosed low ALM by BIA. The specificity was 90%, indicating that 90% of subjects who were diagnosed as normal ALM by DXA were also diagnosed as normal ALM by the BIA.

**Conclusion:**

This comparison showed a poor validity of MF-BIA to assess the absolute values of ALM, but a reasonable sensitivity and specificity to recognize the community-dwelling older adults with the lowest muscle mass.

## Introduction

Approximately 16% of the world population will be older than 65 years in 2050, and the number of persons older than 80 years will increase almost threefold between 2019 and 2050 (1). As society ages, the number of people facing physical disabilities due to co- and multi-morbidity will increase as well. Globally, over 45% of older adults aged 60 and over experience disabilities and physical limitations (2). A key contributor to these physical limitations is the reduction in skeletal muscle mass and strength, also referred to as sarcopenia (3, 4). Sarcopenia is defined as a skeletal muscle disorder that involves the accelerated loss of muscle mass and function (5). Sarcopenia also increases the risk for chronic diseases such as type II diabetes and obesity (6, 7) and is associated with fall incidence, institutionalization, dependence, and poor quality of life (8). Moreover, sarcopenia will increase the demand of our healthcare system and will result in tremendous healthcare costs (9).

To reduce these negative outcomes, screening for sarcopenia in clinical practice is of major importance (10). It allows the professional to identify those people at risk for negative outcomes and to intervene with nutritional and exercise strategies to prevent or counteract sarcopenia (11–14).

To assess sarcopenia, various criteria are proposed (3, 4, 8, 15–17). The majority of those criteria (8, 15–17) use dual-energy X-ray absorptiometry (DXA) to quantify appendicular lean mass (ALM). As such, DXA is considered as the reference standard to assess ALM. In practice, however, DXA is not often used as it is too expensive and needs safety precautions and proper training. In practice, bio-impedance analysis (BIA) is suggested as a practical alternative for DXA to diagnose sarcopenia (7, 8, 10, 16, 18). BIA is cheap, fast, and may provide an easy-to-use tool for professionals for diagnosis (19). The validity of BIA, however, is often discussed (7, 8, 10, 20, 21). Several studies assessed the validity of BIA against DXA only validating fat-free mass (FFM) (22–24). To date, only limited studies are available, which validate the ALM assessment by BIA against DXA (10, 25–29); and even few studies are available, which validate the diagnosis of low ALM by BIA against DXA (27, 30). The use of multi-frequency BIA (MF-BIA) including standing position is, furthermore, scarce with ALM validation (21, 31). Therefore, we aim to validate the assessment of ALM as well as the detection of low ALM by MF-BIA against DXA in older adults.

## Materials and Methods

### Subjects

Baseline data of subjects in the VITAMIN (Vital Amsterdam older adults in the city) study (32, 33) were included in the present analysis. At baseline, the ALM and FFM of all subjects are assessed by both DXA and BIA. These subjects were recruited at community-based weekly exercise programs and by a mailing to community-dwelling inhabitants of Amsterdam and its surroundings. Subjects were included in the VITAMIN trial when they were 55 years or older, were able to understand the Dutch language, and were excluded when they were cognitively impaired [mini-mental state examination (MMSE) < 15], had a knee or hip surgery in the past 6 months, or had current alcohol or drug abuse in the opinion of the investigator. A full description of the eligibility criteria is online available in the Dutch Trial Register (NL5472/NTR5888^1^). The study was approved by the Medical Ethics Committee VUmc, Netherlands (Protocol ID: VUMC2016_025), and the written informed consent was obtained. All assessments were performed at the Amsterdam Nutritional Assessment Center (ANAC) in the Amsterdam University of Applied Sciences (AUAS) in Netherlands.

### Anthropometry

Subjects were asked to come to the baseline visit in a semi-fasted state (5-h fasted, 2-h no drinks). Before the measurement routine, they went to changing room and removed their clothes (including jewelry and removable aids). This protocol provides high accuracy in follow-up measurements (19). Bodyweight was measured on a calibrated scale (Bodpod, Life Measurement Concord, United States). Height was measured to the nearest cm by using a wall-mounted stadiometer (Seca 222; Seca, Germany).

### Bio-Impedance Analysis

The ALM and FFM both were assessed in a semi-fasted state by the Tanita MC-780MA (2015, Tanita Corporation, Japan). This Tanita MC-780MA is an 8 electrode multi-frequency (5 kHz/50 kHz/250 kHz) segmental body composition analyzer that predicts ALM and FFM from resistance and reactance (23). Some research is available on the validity of the device in healthy subjects and patients (34, 35). Impedance measurement includes whole body and limb segments, working with a constant current source (∼90 A) with a high frequency (50 kHz). Anthropometry data concerning the subject (age, gender, and height) were entered by the accessor in the GMon Health Monitor software. By mounting the BIA scale, body weight was measured, and by holding the two handles with arms separated from the trunk, body composition analysis was performed in 1 min. All BIA measurements followed standard operating procedures. ALM-index (ALMi) and FFM-index (FFMi), our secondary parameters besides FFM, were calculated by dividing ALM and FFM by height in meters squared.

### Dual Energy X-Ray Absorptiometry

The body composition was assessed by DXA (Hologic Inc., MA, United States) and the Apex software (version 5.5.3; Hologic Inc., Bedford, MA, United States). ALM and FFM were used for the analyses. ALM is the sum of the FFM minus bone mass in arms and legs; and FFM is the total fat-free mass including bone mass. The DXA was calibrated daily with a phantom. After lying down on the DXA table, the subject’s feet were fixed to ensure a steady and correct position. Additionally, their arms were separated from the trunk by material which does not affect the DXA image and analysis. This procedure improves the segmentation analysis of the DXA image. Successively, the whole body DXA scan was performed in 3 min. If subjects didn’t fit the DXA table because of their body size, one side of the body was duplicated for analysis. The automatic segmentation of the whole body scan, thus arms and legs for ALM, was adjusted manually with the use of Hologic software (36). All the DXA measurements and segmentation analysis were performed by the same two trained and certified researchers [CD, JH] (37, 38).

### Statistical Analysis

The double-data entry was performed and discrepancies were checked and adjusted. The primary parameter was ALM and the secondary parameters were ALMi, FFM, and FFMi. The validity for assessing absolute values of these parameters was evaluated by: (1) the bias, which is the mean difference between the two methods. (2) The percentage of accurate predictions, which is defined as a value within 5% of DXA values [this is consistent with technical measurement errors of 5% or less (39)]. If the BIA value was below 95% of the DXA value, the BIA value was defined as an underestimation, and a value above 105% was defined as an overestimation. (3) The mean absolute error, which is presented to give insight on the average absolute deviation in kg (ALM and FFM) or kg/m^2^ (ALMi and FFMi) between the two methods (40). (4) The limits of agreement analysis (Bland–Altman analysis) and proportional bias.

The lowest quintile (20%) of ALM (and the secondary parameters) by DXA was used as proxy for low ALM. Sensitivity and specificity of diagnosis a low ALM by BIA were assessed. The derived cut-offs from the lowest quintile were processed by cross tables to identify the Sensitivity (e.g., the true positive rate for both low ALM DXA and BIA) and Specificity (e.g., the true negative rate for both normal ALM DXA and BIA). In an additional analysis, subjects that were not classified as low ALM (whereas they were according to DXA) were compared to those that were correctly classified as low ALM to identify potential differences in subject characteristics.

The data analyses were performed for the total group and for men and women separately, since regression analysis with outcomes, FFM and FFMi by DXA showed a significant interaction between gender and FFM and FFMi by BIA (*p* < 0.10). No significant interaction was found between gender and ALM and ALMi, but for consistency reasons, all analyses are both presented for the total group and for men and women separately. Bland –Altman was checked for proportional bias, by evaluation of significant slopes for the regression lines. The statistical analyses were performed using the SPSS software (version 25.0, IBM). Mean absolute errors were calculated in MS Excel 2013.

## Results

### Subject Characteristics

**FIGURE 1:**
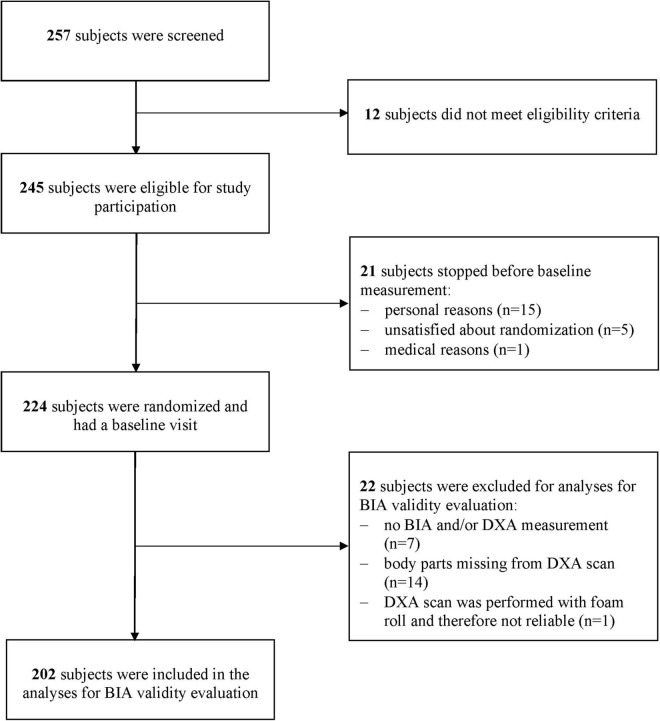
Flow chart of participants for the inclusion in the analysis for BIA validity evaluation.

Of the 224 subjects included in the trial, 202 subjects were used in the present data analysis. Twenty-two subjects could not be included due to missing or poor quality of BIA and/or DXA assessments (Figure 1). The mean age of the subjects was 72.1 ± 6.4 year, with a BMI of 25.4 ± 3.6 kg/m^2^ and 71% were women. Average time of the last drink until the assessment was 7.7 ± 4.9 h. Based on the EWGSOP2, ALMi-cutoffs (8) prevalence of sarcopenic low ALM (assessed by DXA) was 9% in men (5 out of 58) and 10% in women (15 out of 144) (Table 1).

**TABLE 1:** Baseline characteristics of 202 older subjects of the VITAMIN-trial^1^.

	All subjects (*N* = 202)		Males (*N* = 58)		Females (*N* = 144)	
	Mean ± SD	Range^1^	Mean ± SD	Range	Mean ± SD	Range
Age (y)	72.1 ± 6.4	55–89	72.0 ± 6.0	60–89	72.1 ± 6.6	55–88
Low education level (%)^2^	21%		17%		22%	
BMI (kg/m^2^)	25.4 ± 3.6	16.8–39.2	25.2 ± 3.0	20.2–32.8	25.6 ± 3.8	16.8–39.2
Fat percentage (%, by DXA)	32.0 ± 6.4	17.3–46.0	24.9 ± 3.1	19.3–31.9	34.8 ± 4.9	17.3–46.0
Handgrip strength (kg)^3^	29.4 ± 10.6	6.7–66.2	41.3 ± 10.0	23.0–66.2	24.6 ± 6.1	9.8–41.3
Gait speed (m/s)^4^	1.34 ± 0.36	0.48–2.08	1.47 ± 0.43	0.75–2.80	1.29 ± 0.32	0.48–2.13
Waist circumference (cm)	89.1 ± 10.4	65–123	95.0 ± 8.7	79 –112	86.7 ± 10.1	65–123
Waist/hip ratio	0.88 ± 0.08	0.69–1.14	0.96 ± 0.06	0.82–1.14	0.85 ± 0.06	0.69–1.05
ALM (kg, by DXA)	20.4 ± 4.2	13.5–35.3	25.6 ± 3.1	19.1–35.3	18.3 ± 2.4	13.5–25.4
FFM (kg, by DXA)	49.9 ± 8.9	34.5–78.3	60.5 ± 7.8	47.0–78.3	45.6 ± 5.3	34.5–62.3
ALMi (kg/m^2^, by DXA)	7.2 ± 1.0	5.3–11.3	8.2 ± 0.9	6.6–11.3	6.8 ± 0.7	5.3–9.8
FFMi (kg/m^2^, by DXA)	17.7 ± 2.1	13.6–24.2	19.9 ± 1.5	17.2–24.2	16.8 ± 1.6	13.6–22.9
Sarcopenic low ALMi (%)^5^			9%		10%	

*^1^Range is presented in minimum to maximum value.*

*^2^Low education level is defined as highest finished education is primary or secondary school.*

*^3^Average handgrip strength of the dominant hand.*

*^4^Measured by a 3-meter gait speed test, fastest of 2 repetitions.*

*^5^Based on the EWGSOP2 ALMi-cutoffs (male < 7.0 kg/m^2^ | female < 6.0 kg/m^2^).*

### Validity of Bio-Impedance Analysis

The evaluation of the validity of the BIA to assess ALM, ALMi, FFM, and FFMi is presented in Table 2. Overall, BIA slightly underestimated ALM compared to DXA with a mean bias of −0.60 ± 1.21 kg. The percentage of accurate predictions was 54% for all subjects.

**TABLE 2:** Evaluation of the validity of appendicular lean mass (ALM), fat free mass (FFM), ALM-index (ALMi), and FFM-index (FFMi), including sensitivity and specificity of diagnosing low muscle mass, assessed by BIA with DXA as reference^1^ in 202 subjects of 55 years and older.

	ALM (kg)	ALMi (kg/m^2^)	FFM (kg)	FFMi (kg/m^2^)
	All subjects (*N* = 202)			
Reference by DXA [mean (± SD)]	20.4 ± 4.2	7.2 ± 1.0	49.9 ± 8.9	17.7 ± 2.1
Mean (± SD)	19.8 ± 3.8	7.0 ± 0.9	50.1 ± 9.4	17.7 ± 2.1
Mean Bias^2^ (± SD)	−0.60 ± 1.21	−0.20 ± 0.43	0.24 ± 2.31	0.06 ± 0.80
Mean Bias in% (± SD)	−2.5 ± 5.8	−2.5 ± 5.8	0.4 ± 4.6	0.4 ± 4.6
Mean abs. error (kg or kg/m^2^)	1.1	0.4	1.7	0.6
Accurate predictions^3^ (%)	54.0	54.0	77.7	77.7
Under predictions^4^ (%)	37.6	37.6	9.4	9.4
Over predictions^5^ (%)	8.4	8.4	12.9	12.9
Sensitivity%^6^	79.5%	74.4%	76.9%	59.0%
Specificity%^7^	89.6%	84.0%	93.3%	92.6%

	**Males (*N* = 58)**			
Reference by DXA [mean (± SD)]	25.6 ± 3.1	8.2 ± 0.9	60.5 ± 6.8	19.3 ± 1.9
Mean (SD)	24.5 ± 2.9	7.8 ± 0.8	62.5 ± 5.7	19.9 ± 1.5
Mean Bias^2^ (SD)	−1.1 ± 1.2	−0.4 ± 0.4	2.0 ± 2.1	0.6 ± 0.7
Mean Bias in% (SD)	−4.2 ± 4.5	−4.2 ± 4.5	3.6 ± 3.7	3.6 ± 3.7
Mean abs. error (kg or kg/m^2^)	1.3	0.4	2.5	0.8
Accurate predictions^3^ (%)	50.0	50.0	63.8	63.8
Under predictions^4^ (%)	46.6	46.6	0	0
Over predictions^5^ (%)	3.4	3.4	36.2	36.2
Sensitivity%^6^	90.9%	100%	54.5%	36.4%
Specificity%^7^	89.4%	83.0%	100%	100%
Cut-off DXA value^1^ (kg or kg/m^2^)	22.8	7.4	55.0	17.5

	**Females (*N* = 144)**			
Reference by DXA [mean (± SD)]	18.5 ± 2.6	6.9 ± 0.8	46.2 ± 6.1	17.1 ± 1.9
Mean (SD)	18.1 ± 2.3	6.7 ± 0.7	45.7 ± 5.7	16.9 ± 1.8
Mean Bias (SD)^2^	−0.4 ± 1.2	−0.1 ± 1.4	−0.5 ± 2.1	−0.2 ± 0.8
Mean Bias in% (SD)	−1.8 ± 6.2	−1.8 ± 6.2	−0.9 ± 4.4	−0.9 ± 4.4
Mean abs. error (kg or kg/m^2^)	1.0	0.4	1.5	0.5
Accurate predictions^3^ (%)	56.9	56.9	83.3	83.3
Under predictions^4^ (%)	32.6	32.6	13.2	13.2
Over predictions^5^ (%)	10.4	10.4	3.5	3.5
Sensitivity%^6^	75.0%	64.3%	85.7%	67.9%
Specificity%^7^	89.7%	84.5%	90.5%	89.7%
Cut-off DXA value^1^ (kg or kg/m^2^)	16.1	6.2	41.3	15.5

*^1^Low muscle mass diagnosed by ALM, FFM, ALMi or FFMi is defined as the lowest sex-specific quintile of each variable measured by DXA, N in the lowest quintile is 39 subjects: 11 for males and 28 for females.*

*^2^BIA value minus DXA value.*

*^3^The percentage of subjects predicted by this predictive equation within 5% of the DXA value.*

*^4^The percentage of subjects by this predictive equation < 5% of the DXA value.*

*^5^The percentage of subjects predicted by this predictive equation > 5% of the DXA value.*

*^6^Sensitivity is to be interpreted as the percentage of subjects with low ALM according to DXA that also had low ALM by BIA.*

*^7^Specificity is to be interpreted as the percentage of subjects with normal ALM according to DXA also had normal ALM (ALMi, FFM, or FFMi) with the BIA.*

**FIGURE 2:**
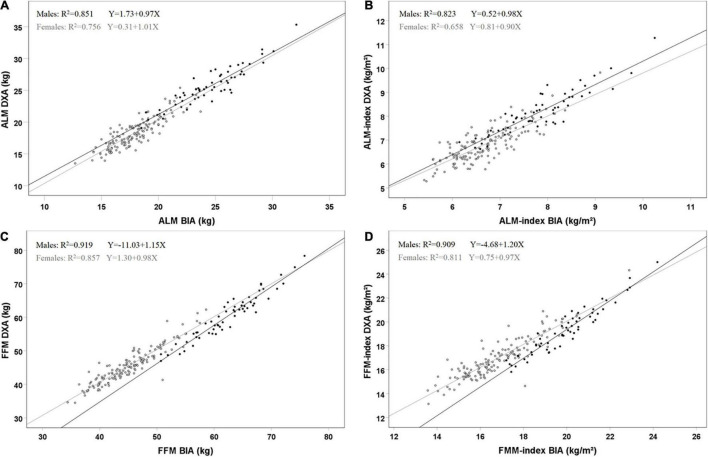
Scatterplots of agreement for BIA and DXA for ALM **(A)**, ALM-index **(B)**, FFM **(C)** and FFM-index **(D)** in 202 subjects (58 males, 144 females). Black dots and lines represent males, and gray dots and lines females. R^2^ and regression lines are reported in the plots. **(A)** males [F(1, 56) = 320.2, *p* < 0.001] and females [F(1, 142) = 439.2, *p* < 0.001]; **(B)** males [F(1, 56) = 260.2, *p* < 0.001] and females [F(1, 142) = 272.8, *p* < 0.001]; **(C)** males [F(1, 56) = 637.0, *p* < 0.001] and females [F(1, 142) = 849.3, *p* < 0.001]; **(D)** males [F(1, 56) = 556.1, *p* < 0.001] and females [F(1, 142) = 611.3, *p* < 0.001].

**FIGURE 3:**
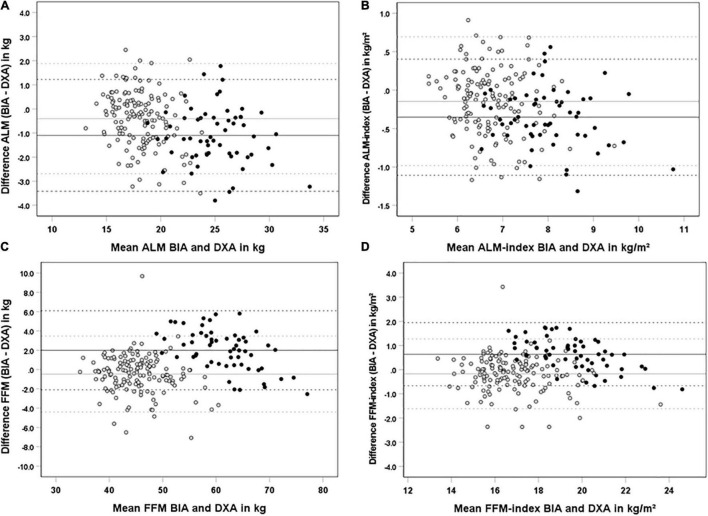
Bland-and-Altman plots for agreement of BIA and DXA for ALM **(A)**, ALM-index **(B)**. FFM **(C)** and FFM-index in kg/m^2^ **(D)** in 202 subjects (58 males. 144 females). Black dots and lines represent males, and gray dots and lines females. Continued lines represent the mean bias, dashed lines represent the limits of agreement [mean ±(1.96 × SD)]. Proportional bias is reported when the slope of the regression line was significant. **(A)** females R^2^ = 0.08 | Y = 2.41–0.16X, *p* < 0.001); **(B)** females R^2^ = 0.03 | Y = 0.63–0.12X, *p* = 0.035); **(C)** males R^2^ = 0.28 | Y = 13.1–0.18, *p* < 0.001); **(D)** males R^2^ = 0.38 | Y = 5.29–0.24, *p* < 0.001.

Scatter plots and Bland–Altman plots in Figures 2, 3 visualize the agreement between BIA and DXA for all parameters. The limits of agreement for ALM were −3.0 to + 1.8 kg. The Bland–Altman analysis shows proportional bias for women in ALM, with a significant negative β for BIA–DXA difference compared to the mean of BIA and DXA [ALM women β = −0.16 (*SE* = 0.04), *p* < 0.001]. This indicates a great underestimation by BIA for larger values of ALM in women, and overestimation appears in the smaller values of ALM. No significant proportional bias for men was present for ALM. See Figure 3 for additional results on the proportional bias.

### Sensitivity and Specificity for Diagnosis of Sarcopenia

Sensitivity and specificity percentages for all parameters are presented in Table 2. In total 39 subjects (11 men and 28 women) were defined as low ALM by using the lowest quintile of ALM by DXA as cut-off. Sensitivity for detecting low ALM using by BIA was 80%, indicating that 80% of subjects that had low ALM according to DXA were also diagnosed as low ALM by BIA. Specificity was 90%, indicating that 90% of subjects who were diagnosed as normal ALM by DXA were also diagnosed as normal ALM by the BIA.

In an additional analysis, subjects that were incorrectly classified as low ALM were compared to those that were correctly classified as low ALM to identify potential differences in subject characteristics. A total of 7 out of 28 women were incorrectly diagnosed as normal ALM by BIA (while DXA diagnosed them as low ALM). These women had a significantly higher BMI (25.5 ± 3.9 *vs*. 22.4 ± 2.5 kg/m^2^, *p* = 0.026) and fat percentage (37.6 ± 6.1 *vs*. 33.2 ± 4.3%, *p* = 0.044) than women that were correctly diagnosed as low ALM by BIA (21 out of 28) (see Supplementary Figure 1). For men, this analysis could not be performed, since only 1 male (1 out of 11) was incorrectly diagnosed as low ALM by BIA.

## Discussion

In the present analyses, the validity of BIA against the DXA was assessed to quantify ALM and diagnose low ALM in community-dwelling older adults. Results showed a poor validity of BIA to assess absolute values of ALM, since the number of accurate predictions was low. Sensitivity and specificity for low ALM were reasonable and superior for ALM compared to ALMi, FFM, and FFMi. Additional analysis with a small sample revealed more overweight women as prone to be misdiagnosed as normal ALM while having low ALM, which is relevant for the assessment of sarcopenic obesity.

Sarcopenia, of which a low ALM is a key component, is known to markedly increase the risk of disability and loss of functional capacity and worsen clinical outcomes in older adults. To reduce these negative outcomes, screening for sarcopenia is of great importance (8), since the progress of sarcopenia can be slowed down or even reversed (41). ALM is needed to diagnose sarcopenia according to most definitions (4, 8, 15–17). Until now, several studies evaluated the validity of ALM by BIA in older adults (10, 24, 26–28). Contradictory to our study, most of these studies developed a new predictive equation by using the crude resistance and reactance values from the BIA device (10, 24, 26, 28). These studies did not evaluate the ability to diagnose sarcopenic low ALM in older adults. To our knowledge, only three studies are available that evaluated the sensitivity in diagnosing sarcopenia (27, 30, 42). Steihaug et al. (27) compared the validity of four BIA equations in estimating ALM in an older population 3 months after hip fracture. They showed sensitivity for estimating low ALM mass according to EWGSOP criteria (43) of 32–84% in men and 42–66% in women depending on the equation used. The sensitivity to assess sarcopenia in their study was lower for all equations compared to the sensitivity we found for the assessment of low ALM with the MF-BIA Tanita MC-780 (men 91%, women 75%). Both studies, Reiss et al. (30) and Sousa-Santos et al. (42) compared the DXA–BIA approaches with EWGSOP/EWGSOP2 cut-offs, and reported the accuracy in older adults. Although the sensitivity seemed comparable [55–70% in estimating reduced muscle mass, 69–92% in estimating sarcopenia (30), and 33–92% in diagnosing sarcopenia (42)], the methods differed. They handled with formulas and cut-offs as a reference, whereas we used the lowest quintile as reference. As well as gender was not taken into account. In general, the observed differences between studies might be attributed to the differences in cut-offs, device used (single/multi-frequency), position and the older population characteristics (42).

Regarding the device used, the study of Kim et al. (31) is the only comparable study with MF-BIA (InBody 720 *vs*. our Tanita MC-780MA) in older adults. Similar to our study, the authors found an overestimation of FFM and they found underestimation of whole-body LM. So far, our study is the only one that evaluates the validity of estimating absolute values of ALM and the ability to diagnose low ALM by MF-BIA in community-dwelling healthy older adults.

As mentioned by Scafoglieri et al. and Walowski et al. (28, 44), BIA models have the tendency to overestimate ALM in sarcopenic older adults, and therefore, underestimate sarcopenia in obesity. Recent research confirms this overestimation of obese adults (45). Our study supports this, furthermore, our additional analysis revealed misdiagnosis among overweight women. This result needs to be interpreted with caution, because of the small sample. Nevertheless, a more extensive analysis of overweight older adults with a larger sample seems interesting for future research.

It should be noted that when comparing two methods, as is the case in all validation studies, there are always measurement errors in both methods. Not only measurement errors occur with the BIA, but the DXA has also measurement errors depending on the thickness of the tissue measured, the hydration status, and the calibration procedures (46). Furthermore, for measuring ALM with the DXA, a correct adjustment of the automatic segmentation has to be performed manually, which may introduce a measurement error. Since we use the DXA as the reference value, we can only demonstrate relative validity compared to DXA. Although magnetic resonance imaging (MRI) and computed tomography (CT) are regarded as the golden standard, recent literature suggests that the DXA can be used as a reference to measure muscle mass (47).

### Study Limitations

Some study limitations are important to mention: First, we studied a relatively healthy population of community-dwelling older adults. Subjects were recruited at community-based weekly exercise programs, so the population was performing exercise at least once a week. Therefore, our definition of sarcopenic low ALM based on the lowest quintile of ALM (or FFM, ALMi, or FFMi) was less strict compared to existing definitions for sarcopenia (4, 8, 15–17). Our cut-off DXA value *vs*. the EWGSOP2 (8) was slightly higher for men (22.8 *vs*. 20.0 kg) and for women (16.1 *vs*. 15.0 kg). At the lower end of ALM values, there were only a limited number of outliers and a fairly gradual increase across the whole ALM value range. Therefore, we do not expect a more strict 10% cut-off for low ALM to provide other results than this 20% cut-off. However, we can be more sure for women than men. Second, the overall sample size and the sample size for women are sufficient to perform the presented analysis, but the number of men in our study was low (48). Last, for this evaluation we used the formula of the Tanita MC-780MA itself, we did not use an existing validated equation based on measured resistance and reactance for a comparable population. Using the most appropriate equation among the equations available in the literature, taking into account nutritional, ethnic-related, and age-related characteristics of the sample in which the equation has been validated, might even improve the sensitivity of low ALM diagnosis (26).

Multi-frequency bio-electrical impedance analysis is a cheap and quick devise and very easy to use in practical settings (19). However, our results show that the assessment of absolute values of ALM has to be interpreted with caution due to the low accuracy of prediction. Only just over 50% of our study population was correctly estimated. This study did not focus on the validity to detect changes over time. To evaluate the effectiveness of interventions aiming at preventing or counteracting sarcopenia, future studies should focus on the validity of the MF-BIA in detecting changes in ALM. For diagnosis of low ALM, the Tanita MC-780MA MF-BIA seems reasonably accurate, but future studies need to confirm this in a population with lower muscle mass. Finally, future studies might include large samples of older adults in order to find optimal cut-offs for diagnosing low ALM by MF-BIA and support clinical practice.

In conclusion, our comparison showed a poor validity of this MF-BIA to assess absolute values of ALM, but a reasonable sensitivity and specificity to recognize the community-dwelling older adults with the lowest muscle mass. Regardless of reaching the sarcopenic cut-offs, the recognition of low ALM is an important step toward prevention of sarcopenia.

## Data Availability Statement

The datasets presented in this study can be found in online repositories. The names of the repository/repositories and accession number(s) can be found below: DOI: 10.21943/auas.19165094.

## Ethics Statement

The studies involving human participants were reviewed and approved by METc VU University Medical Center. The patients/participants provided their written informed consent to participate in this study.

## Author Contributions

PW, MT, and JH designed the research (project conception, development of overall research plan, and study oversight). JH and CD conducted the research (hands-on conduct of the experiments and data collection). AV, JH, ME, PW, and MT analyzed the data or performed the statistical analysis. AV, JH, ME, PW, and MT wrote the manuscript. MT and PW had primary responsibility for the final content. All authors contributed to the article and approved the submitted version.

## Conflict of Interest

The authors declare that the research was conducted in the absence of any commercial or financial relationships that could be construed as a potential conflict of interest.

## Publisher’s Note

All claims expressed in this article are solely those of the authors and do not necessarily represent those of their affiliated organizations, or those of the publisher, the editors and the reviewers. Any product that may be evaluated in this article, or claim that may be made by its manufacturer, is not guaranteed or endorsed by the publisher.

## Footnotes

1
www.trialregister.nl
