# Supplementary material for: Bio-Electrical Impedance Analysis: A Valid Assessment Tool for Diagnosis of Low Appendicular Lean Mass in Older Adults?
Source: Front Nutr. 2022 Jun 2;9:874980. doi: 10.3389/fnut.2022.874980 (PMC9201397; doi:10.3389/fnut.2022.874980)
Supplement: Supplementary file 1 [file Image_1.pdf]

Supplementary Material Presentation

1 Supplementary Figures

1.1 Additional Figure 1

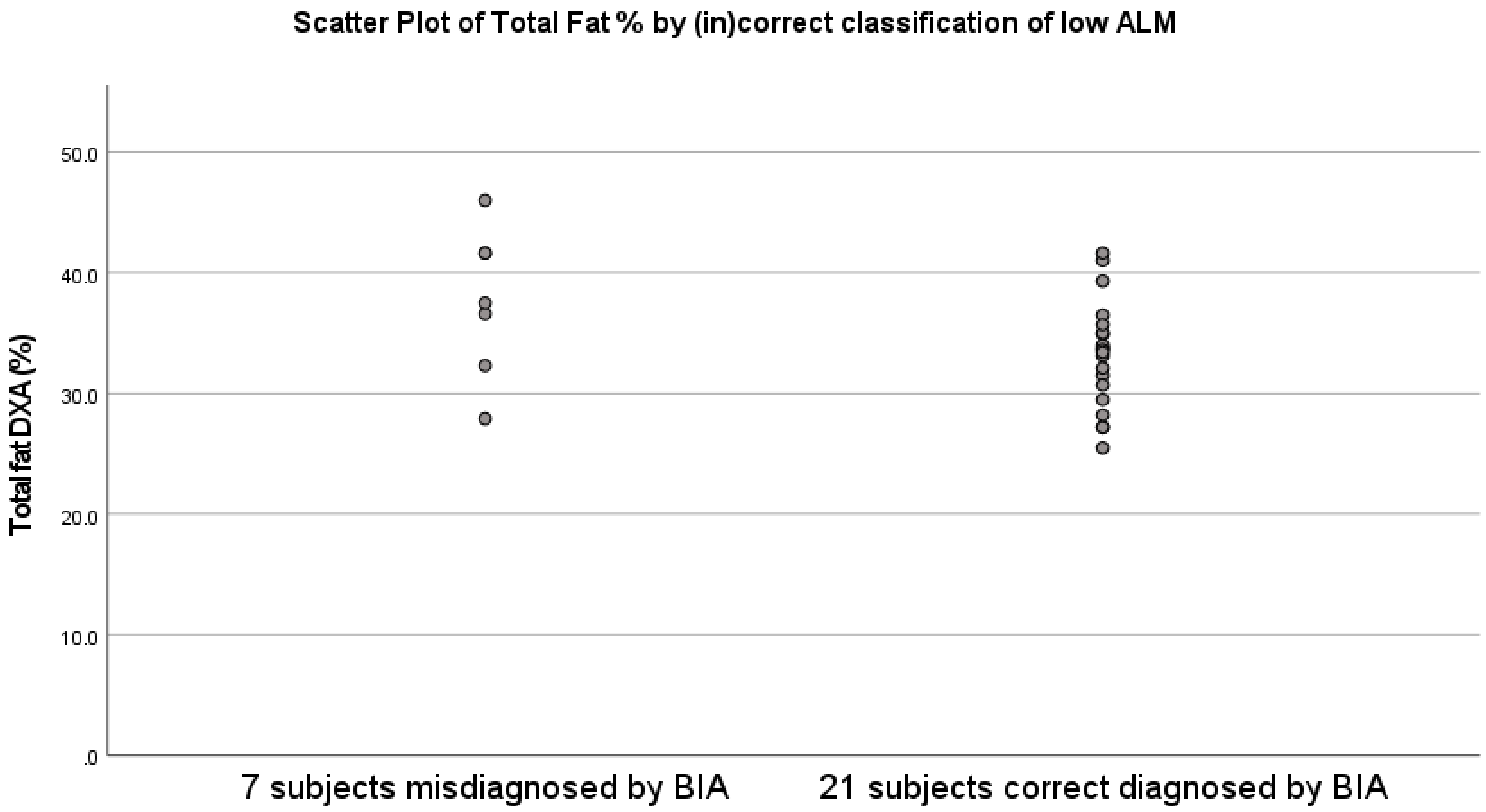

Additional Figure 1: Scatter plot of total fat percentage for the 28 females categorized as low ALM. 7 subjects that were misdiagnosed by BIA (normal for BIA, low ALM for DXA) have a significant higher total fat percentage, compared to the 21 correctly diagnosed subjects with low ALM. (37.6±6.1 vs. 33.2±4.3 %, p=0.044). The dots represent females.
